# Speed of Retinal Vascularization in Retinopathy of Prematurity: Risk and Protective Factors

**DOI:** 10.1155/2019/2721578

**Published:** 2019-04-24

**Authors:** Ana Maria Solans Pérez de Larraya, José María Ortega Molina, José Uberos Fernández, Amanda Rocío González Ramírez, José Luis García Serrano

**Affiliations:** ^1^Department of Ophthalmology, San Cecilio University Hospital, Granada, Spain; ^2^Department of Paediatrics, San Cecilio University Hospital, Granada, Spain; ^3^Biomedical Research Institute, ibs. GRANADA, University Hospitals in Granada, University of Granada, Spain

## Abstract

**Objective:**

The objective was to study the risk and protective factors involved in retinal vascular development of preterm infants with retinopathy of prematurity.

**Methods:**

Between 2000 and 2017, 185 preterm infants were included in the protocol for retinopathy of prematurity. Risk factors associated with speed of retinal vascularization <0.5 disc diameter/week were studied in each of them.

**Results:**

The statistically significant variables related to retinal vascular development <0.5 DD/w were intubation days, degree 3 of bronchopulmonary dysplasia, weight gain at 4-6 weeks, avascular temporal area, gestational age, number of transfusions, sepsis, number of risk factors, apnea at birth, presence of ductus arteriosus, and days of continuous positive airway pressure therapy. After the multivariate logistic regression analysis, only three variables were found to be significant: intubation days (p=0.005), degree 3 of bronchopulmonary dysplasia (p=0.022), and weight gain at 4–6 weeks (p=0.031).

**Conclusion:**

In retinopathy of prematurity, degree 3 of bronchopulmonary dysplasia and intubation days cause delayed retinal vascular development, whereas greater postnatal weight gain favors an appropriate rate of retinal vascularization.

## 1. Introduction

Retinopathy of prematurity (ROP) is a vasoproliferative disease of multifactorial etiology due to abnormal vascularization of the retina. The more advanced the stage of ROP is, the more vascular and ischemic changes are found and therefore more weeks are required for completion of vascularization and subsequent involution [1]. Mintz measured the retinal vascular development in disc diameters per week (DD/w). The speed of vascularization was found to be lower in patients with recurrent ROP than in those without recurrence [2].

The higher the stage of ROP is, the lower the speed of vascularization will be. Preterm infants with delayed or insufficient retinal vascularization have an increased risk for ROP that can be treated with laser and/or anti-VEGF.

This risk increases when the temporal retinal vascularization is lower than 0.5 disc diameter/week (DD/w) [3].

The purpose of this study was to analyze the risk and protective factors associated with the speed of retinal vascularization in those premature infants included in the ROP protocol.

## 2. Material and Methods

Between 2000 and 2017 we explored a total of 612 premature infants in San Cecilio University Hospital of Granada, Spain, following our hospital protocol for ROP, of which 417 were excluded from the sample because they did not meet the inclusion criteria. A total of 185 babies were eligible to take part in the study. Clinical data were collected retrospectively from the inclusion of premature infants in said protocol to the medical discharge. ROP was recorded according to the International classification of ROP.

The inclusion criteria were the following: to be born with a gestational age of ≤32 weeks, to undergo at least three examinations, and to be diagnosed with stages 1, 2, or 3 of ROP. The exclusion criteria were < 3 explorations, stages 4, 5, and aggressive ROP (APROP) (low number of cases), opacity of means, and congenital ophthalmological or systemic abnormalities [3].

The degree of retinopathy in each eye was determined via indirect ophthalmoscopy with indentation and after pharmacological mydriasis by an expert ophthalmologist in this technique, using for this purpose a 20-diopter lens. This lens provides a magnification of 2.5, allowing a 45° view of the retina, the equivalent of 8 disc diameters. Speed of temporal retinal vascularization (DD/w) was calculated as the ratio between the extent of temporal retinal vascularization (DD) and the time in weeks (w). The horizontal disc diameter (DD) was used as a unit of length [3].

The speed of temporal retinal vascularization <0.5 DD/w was considered a dependent variable. Predictive factors included intubation days, presence or absence of degree 3 of dysplasia bronchopulmonary (BPD 3), weight gain (gr/day) at 4–6 weeks, avascular temporal retinal area (DD), gestational age (weeks), performance of transfusions, presence or absence of sepsis, number of risk factors, presence or absence of apnea at birth, patent ductus arteriosus, and days with continuous positive airway pressure (CPAP) therapy.

Sepsis is defined as life-threatening organ dysfunction caused by a dysregulated host response to infection [4]. All the patients showed positive results in the fungal and/or bacterial culture as well as hematological disorders compatible with sepsis.

Apnea is defined as cessation of breathing for at least 10 seconds; a duration of at least 20 seconds is frequently used to diagnose significant apneas, as normal infants do not have apneas of greater than 20 seconds [5].

BPD is a chronic lung disease that causes decreased alveolar and capillary development, resulting in limitations of the respiratory function [6, 7]. It can be classified into three different severity grades. The presence of any degree of BPD is recorded. According to the thresholds proposed by NIHCD and by Jobe and Bancalari, BPD is defined as a need for supplemental oxygen >21% at 28 days of life and/or a need for supplemental oxygen >21% or for positive airway pressure at 36 weeks' corrected gestational age. BPD is classified as mild, moderate, or severe [8, 9].

Between 2000 and 2017, oxygenation protocols were modified. During the years 2000 and 2009 oxygen saturation levels were maintained around 95%; from 2009 oxygen saturation target decreased between 85 and 93% [10].

Statistical Package for Social Sciences (SPSS 15.0, Inc., Chicago, IL) was used for the statistical analysis. The data were analyzed using descriptive statistics and *χ* ^2^ testing. Subsequently, a binary logistic regression analysis was performed to determine the risk factors influencing the vascularization <0.5DD/w. Risk factors were analyzed through univariate analysis and then by applying the bivariate logistic regression model. The independent variables or risk factors considered in our study are described in Table 1. The odds ratio (OR) with 95% CIs associated with each predictor was calculated from logistic regression models. The final model obtained contains risk factors with p<0.05.

## 3. Results

### 3.1. Demographic Characteristics of the Study Population

From the total of 185 premature infants, there were 91 (49.2%) males and 94 (50.8%) females. The mean gestational age and birth weight were 28.95 weeks (SD 1.99) and 1119.35 gr (SD 270.94), respectively.

#### 3.1.1. Risk Factors Leading to Speed of Temporal Retinal Vascularization < 0.5 DD/w

A univariate analysis of factors associated with speed of vascularization <0.5DD/w was performed. Table 1 contains the risk factors considered to be statistically significant after the analysis.

#### 3.1.2. Binary Logistic Regression Analysis of Factors Associated with Speed of Retinal Vascularization <0.5 DD/w or ≥ 0.5 DD/w

According to the results of the logistic regression, this model explains 22.5% of the variability of data collected about the speed of vascularization of the patients. Multiple stepwise regression analyses considered to be reliable showed three independent risk factors associated with delayed vascularization speed (Table 2): intubation days [OR: 1.05 (CI: 1.015–1.085), p=0.005], BPD 3 [OR: 2.24 (CI: 1.12–4.48), p=0.022], and weight gain (gr/day) [OR: 0.93 (CI: 0.876–0.994), p=0.031].

The risk is calculated with the following formula:

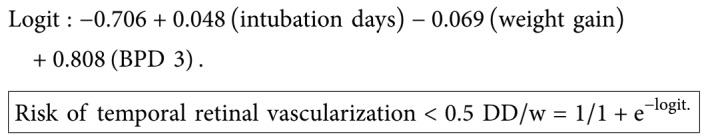
(1) In the model, the factors that cause delayed retinal vascularization in ROP are intubation days and presence of BPD 3. However, weight gain at 4–6 weeks acts as a protective factor.

## 4. Discussion

In severe ROP there is a delay in retinal vascularization [2, 11]. Ashton estimates the normal speed of retinal vascularization in the premature infants to be 0.1mm/day equivalent to 0.7 DD/w [12]. According to our results, premature infants with a vascularization rate < 0.5 DD/w had a greater avascular area at similar gestational age than those with vascularization ≥ 0.5 DD/w; this delay in vascularization significantly increased the need for treatment [3].

Multiple factors have been identified to affect the development of ROP, many of them related to inflammatory mechanisms. Premature infants suffer from delayed retinal vascular development. The delayed retinal vascular development together with other risk factors such as intubation days, BPD ≥ 3, low weight gain at 4-6 weeks, avascular temporal retinal area, very low gestational age, performance of transfusions, sepsis, apnea at birth, PDA, and days of CPAP therapy increases the risk of suffering from severe ROP [13].

The postnatal environment influences premature and more immature infants for longer, adversely affecting the development of retinal vascularization [14]. Other studies describe blood transfusion, the presence of sepsis, apnea, and ductus arteriosus as risk factors for severe ROP; in our work we found statistically significant results for the same factors [15, 16]. Low gestational age and lower birth weight have also been described as predictors of abnormal retinal vascularization [17]. Our results indicate that lower gestational age and lower weight gain delay retinal vascularization.

According to the CRYO-ROP group, patients with ROP in zone I versus patients with ROP in zone II had a higher risk of ROP with unfavourable structural results. The abnormal formation of vessels is greater in those patients with greater avascular area; this determines a delay in the rate of vascularization [18]. The final stage of ROP was found to be less severe when the speed of retinal vascularization was higher [3]. Other authors also found a difference in the speed of vascularization between patients treated with bevacizumab monotherapy with ROP recurrence (0.11 DD/w) and patients without recurrence (0.23 DD/w) [2]. Although the univariate analysis showed that numerous factors are associated with a delay in the speed of retinal vascular development, this study intends to identify the factors causing the temporal development of the retina to be <0.5 DD/w. The only three factors considered to be significant in the multivariate analysis were incubation days, BPD 3, and weight gain at 4-6 weeks.

Many studies indicate that BPD is associated with the development of ROP. High oxygen administration in ROP is a factor that inhibits or delays retinal vascularization [19]. Holmström et al. showed that BPD precedes ROP and acts as a risk factor for its development and severity [20]. Other authors support this theory since premature infants with BPD require oxygen therapy, which may increase the incidence of ROP [21–24]. These results are consistent with other researches confirming that long-term mechanical ventilation and/or CPAP are risk factors for the development of severe forms of ROP [15, 25, 26]. The SUPPORT group found that premature infants with oxygen saturations between 85 and 92% in the first 33 weeks had less delay in vascularization and less risk of severe ROP than patients with higher oxygen saturations [27].

BPD is characterized by abnormal alveolar microvascular development and altered alveolarization. The factors that dysregulate angiogenesis are common to both ROP and BPD [23, 28]. The results of our study showed that the intubation days and BPD 3 were risk factors associated with ROP. However, BPD caused delayed retinal vascular development regardless of the intubation time.

It has been described that adequate energy intake facilitates growth and reduces morbidity. This is consistent with our results because the greater the weight gain, the lower the risk of BPD and ROP [29]. Increased weight gain at 4-6 weeks' postnatal age acts as a protective factor of ROP as it allows for appropriate speed of retinal vascularization.

The developmental period of ROP can be shortened by faster vascularization of the retina. In this study we observe that the factors that influence the delay in vascularization and therefore increase the risk of severe ROP are degree 3 of bronchopulmonary dysplasia and intubation days whereas greater postnatal weight gain favours an appropriate rate of retinal vascularization.

## 5. Limitations

The authors who studied the extension of retinal vascularization between 2014 and 2019 do so on fundus photography or by fluorescein angiography, where they have been able to repeat measurements in a masked manner. The explorations in our work were performed by binocular ophthalmoscope only by a paediatric ophthalmologist on awake children, with the difficulty that entails. The study covers a long period of time, between 2000 and 2017; oxygenation protocols in premature infants were modified so that not all premature infants were treated with the same oxygen therapy protocols, as discussed in Section 2.

In conclusion, the rate of vascularization should be an important factor in the exploration of children with ROP. A speed of retinal vascularization <0.5DD/w should alert us to the increased risk of severe ROP. An increased weight gain at 4-6 weeks' postnatal age is considered as a protective factor in the speed of retinal vascularization in ROP whereas BPD 3 and greater number of intubation days act as risk factors for delayed vascular development.

## Figures and Tables

**Table 1:** Univariate analysis of factors that modify significantly the retinal vascular development: intubation days (d), BPD 3, weight gain at 4-6 weeks (gr/days), avascular temporal area (DD), gestational age (weeks), sepsis, apnea at birth, ductus arteriosus, and CPAP therapy (days).

Risk factor	Odds ratio (CI 95%)	P-value R^2^	Nagelkerke
Intubation days (d)	1.068 (1.03-1.10)	<0.01	15.9%
BPD 3	1.57 (1.2-2.07)	0.01	8.6%
Weight gain (gr/day)	0.92 (0.86-0.97)	0.04	6.9%
Avascular temporal area	1.325 (1.1-1.59)	0.003	6.7%
Gestational age	0.79 (0.68-0.93)	0.04	6.2%
Transfusions number	1.80 (1.12-2.89)	0.015	4.9%
Sepsis	2.21 (1.19-4.1)	0.012	4.7%
Number of risk factors	1.14 (1.02-1.27)	0.024	3.9%
Apnea at birth	2.54 (1.12-5.75)	0.025	3.7%
Ductus arteriosus	2.36 (1.08-5.15)	0.032	3.4%
Days of CPAP therapy	1.05 (1.003-1.10)	0.039	3.2%

**Table 2:** The multiple logistic regression analysis showed 3 independent risk factors leading to speed of vascularization <0.5 DD/w, which were included in the model.

	B	Sig.	Exp (B)	95% CI for EXP (B)
Intubation days	0.048	0.005	1.049	1.015 - 1.085
Weight gain	- 0.069	0.031	0.933	0.876 – 0.994
BPD 3	0.808	0.022	2.244	1.123 – 4.483
Constant	- 0.706	0.121	0.494	

## Data Availability

The data used to support the findings of this study are available from the corresponding author upon request.
